# (*E*)-*N*′-(5-Chloro-2-hydroxy­benzyl­idene)-3,5-dihydroxy­benzohydrazide mono­hydrate

**DOI:** 10.1107/S1600536809007788

**Published:** 2009-03-11

**Authors:** Sa Deng, Ling Han, Shan-shan Huang, Hou-li Zhang, Yun-peng Diao, Ke-xin Liu

**Affiliations:** a College of Pharmacy, Dalian Medical University, Liaoning 116044, People’s Republic of China; b Liao Ning Benxi Third Pharmaceuticals Co Ltd, Liaoning 117004, People’s Republic of China

## Abstract

In the title compound, C_14_H_11_ClN_2_O_4_·H_2_O, the dihedral angle between the two benzene rings is 8.5 (2)° and an intra­molecular O—H⋯N hydrogen bond is observed in the Schiff base mol­ecule. In the crystal structure, the water mol­ecule accepts an N—H⋯O hydrogen bond and makes O—H⋯O hydrogen bonds to two further Schiff base mol­ecules. Further inter­molecular O—H⋯O hydrogen bonds lead to the formation of layers parallel to the *bc* plane.

## Related literature

For background to the synthesis of Schiff base compounds, see: Herrick *et al.* (2008 ▶); Suresh *et al.* (2007 ▶). For the biological activity of Schiff base compounds, see: Bhandari *et al.* (2008 ▶); Sinha *et al.* (2008 ▶). For a related structure, see: Jiang *et al.* (2008 ▶). For bond-length data, see: Allen *et al.* (1987 ▶).
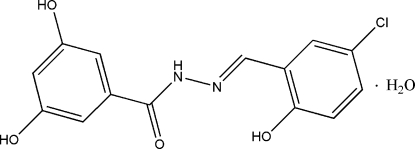


## Experimental

### 

#### Crystal data

C_14_H_11_ClN_2_O_4_·H_2_O
*M* *_r_* = 324.71
Monoclinic, 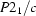

*a* = 14.106 (3) Å
*b* = 8.0090 (16) Å
*c* = 13.127 (3) Å
β = 108.26 (3)°
*V* = 1408.3 (6) Å^3^
*Z* = 4
Mo *K*α radiation
μ = 0.30 mm^−1^
*T* = 298 K
0.20 × 0.20 × 0.18 mm

#### Data collection

Siemens SMART CCD diffractometer
Absorption correction: multi-scan (*SADABS*; Siemens, 1996 ▶) *T*_min_ = 0.943, *T*_max_ = 0.948
6975 measured reflections
2496 independent reflections
1437 reflections with *I* > 2σ(*I*)
*R*_int_ = 0.084

#### Refinement

*R*[*F* ^2^ > 2σ(*F* ^2^)] = 0.053
*wR*(*F* ^2^) = 0.142
*S* = 1.04
2496 reflections
202 parameters
H-atom parameters constrained
Δρ_max_ = 0.28 e Å^−3^
Δρ_min_ = −0.30 e Å^−3^

### 

Data collection: *SMART* (Siemens, 1996 ▶); cell refinement: *SAINT* (Siemens, 1996 ▶); data reduction: *SAINT*; program(s) used to solve structure: *SHELXS97* (Sheldrick, 2008 ▶); program(s) used to refine structure: *SHELXL97* (Sheldrick, 2008 ▶); molecular graphics: *SHELXTL* (Sheldrick, 2008 ▶); software used to prepare material for publication: *SHELXTL*.

## Supplementary Material

Crystal structure: contains datablocks global, I. DOI: 10.1107/S1600536809007788/hb2921sup1.cif

Structure factors: contains datablocks I. DOI: 10.1107/S1600536809007788/hb2921Isup2.hkl

Additional supplementary materials:  crystallographic information; 3D view; checkCIF report

## Figures and Tables

**Table 1:** Hydrogen-bond geometry (Å, °)

*D*—H⋯*A*	*D*—H	H⋯*A*	*D*⋯*A*	*D*—H⋯*A*
O1—H1⋯N1	0.82	1.98	2.685 (4)	144
O1—H1⋯O5	0.82	2.47	2.952 (4)	119
O3—H3⋯O1^i^	0.82	2.10	2.916 (4)	173
O4—H4⋯O2^ii^	0.82	1.99	2.762 (4)	158
N2—H2⋯O5^ii^	0.86	2.09	2.931 (4)	164
O5—H5*A*⋯O2	0.85	1.91	2.760 (4)	174
O5—H5*B*⋯O4^iii^	0.85	2.11	2.902 (4)	156

Symmetry codes: (i) 
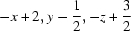
; (ii) 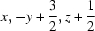; (iii) 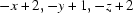.

**Fig. 1.**
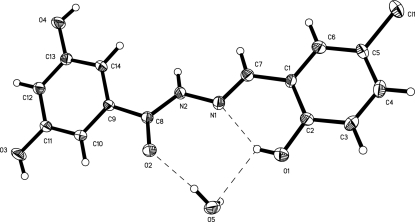
The molecular structure of (I), with displacement ellipsoids drawn at the 30% probability level. The dashed lines indicate hydrogen bonds.

**Fig. 2.**
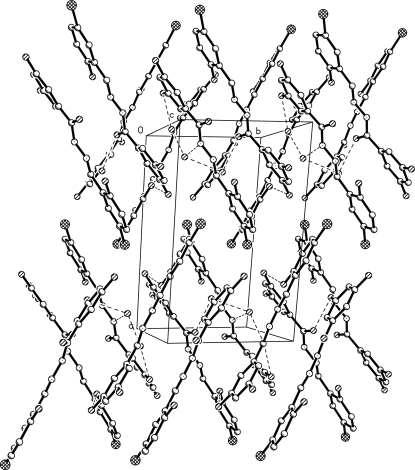
The molecular packing of (I). The donor···acceptor contacts for the intermolecular hydrogen bonds are shown as dashed lines. H atoms are omitted for clarity.

## supplementary crystallographic information

### Comment

Schiff base compounds can be easily synthesized from the reaction of aldehydes with primary amines (Herrick *et al.*, 2008; Suresh *et al.*, 2007). These compounds show interesting biological activities, especially antimicrobial activities (Bhandari *et al.*, 2008; Sinha *et al.*, 2008). In this paper, the crystal structure of the title compound, (I), containing a new Schiff base compound derived from the condensation reaction of 5-chlorosalicylaldehyde with 3,5-dihydroxybenzoic acid hydrazide is reported.

The Schiff base molecule of (I) displays a *trans* configuration with respect to the C=N and C—N bonds (Fig. 1). All the bond lengths are within normal ranges (Allen *et al.*, 1987), and are comparable to those in the related compound 3,5-dihydroxy-*N'*-(2-hydroxybenzylidene) benzohydrazide monohydrate (Jiang *et al.*, 2008). The Schiff base molecule is nearly planar, the dihedral angle between the two benzene rings is 8.5 (2)°. An intramolecular O—H···N hydrogen bond is observed. In the crystal structure the water molecule links three symmetry related molecules through O—H···O and O—H···N hydrogen bonds (Table 1). Together with two further intermolecular O—H···O hydrogen bonds, layers parallel to the *bc* plane are formed (Fig. 2).

### Experimental

5-Chlorosalicylaldehyde (0.1 mmol, 15.6 mg) and 3,5-dihydroxybenzoic acid hydrazide (0.1 mmol, 16.8 mg) were dissolved in a 95% ethanol solution (10 ml). The mixture was stirred at room temperature to give a clear solution. Light yellow blocks of (I) were formed by gradual evaporation of the solvent over a period of nine days at room temperature.

### Refinement

All H atoms were placed in geometrically idealized positions (C—H = 0.93 Å, O—H = 0.82–0.85 Å and N—H = 0.86 Å) and refined as riding with  *U*_iso_(H) = 1.2*U*_eq_(C,*N*) or 1.5*U*_eq_(O).

### Crystal data

**Table:**

C_14_H_11_ClN_2_O_4_·H_2_O	*F*(000) = 672
*M**_r_* = 324.71	*D*_x_ = 1.531 Mg m^−^^3^
Monoclinic, *P*2_1_/*c*	Mo *K*α radiation, λ = 0.71073 Å
Hall symbol: -P 2ybc	Cell parameters from 575 reflections
*a* = 14.106 (3) Å	θ = 3.1–20.4°
*b* = 8.0090 (16) Å	µ = 0.30 mm^−^^1^
*c* = 13.127 (3) Å	*T* = 298 K
β = 108.26 (3)°	Block, light yellow
*V* = 1408.3 (6) Å^3^	0.20 × 0.20 × 0.18 mm
*Z* = 4	

### Data collection

**Table:**

Siemens SMART CCD diffractometer	2496 independent reflections
Radiation source: fine-focus sealed tube	1437 reflections with *I* > 2σ(*I*)
graphite	*R*_int_ = 0.084
ω scans	θ_max_ = 25.0°, θ_min_ = 1.5°
Absorption correction: multi-scan (*SADABS*; Siemens, 1996)	*h* = −16→13
*T*_min_ = 0.943, *T*_max_ = 0.948	*k* = −9→7
6975 measured reflections	*l* = −15→15

### Refinement

**Table:**

Refinement on *F*^2^	Primary atom site location: structure-invariant direct methods
Least-squares matrix: full	Secondary atom site location: difference Fourier map
*R*[*F*^2^ > 2σ(*F*^2^)] = 0.053	Hydrogen site location: inferred from neighbouring sites
*wR*(*F*^2^) = 0.142	H-atom parameters constrained
*S* = 1.04	*w* = 1/[σ^2^(*F*_o_^2^) + (0.0453*P*)^2^ + 1.0153*P*] where *P* = (*F*_o_^2^ + 2*F*_c_^2^)/3
2496 reflections	(Δ/σ)_max_ < 0.001
202 parameters	Δρ_max_ = 0.28 e Å^−^^3^
0 restraints	Δρ_min_ = −0.30 e Å^−^^3^

### Special details

**Table:**

Geometry. All e.s.d.'s (except the e.s.d. in the dihedral angle between two l.s. planes) are estimated using the full covariance matrix. The cell e.s.d.'s are taken into account individually in the estimation of e.s.d.'s in distances, angles and torsion angles; correlations between e.s.d.'s in cell parameters are only used when they are defined by crystal symmetry. An approximate (isotropic) treatment of cell e.s.d.'s is used for estimating e.s.d.'s involving l.s. planes.
Refinement. Refinement of *F*^2^ against ALL reflections. The weighted *R*-factor *wR* and goodness of fit *S* are based on *F*^2^, conventional *R*-factors *R* are based on *F*, with *F* set to zero for negative *F*^2^. The threshold expression of *F*^2^ > σ(*F*^2^) is used only for calculating *R*-factors(gt) *etc*. and is not relevant to the choice of reflections for refinement. *R*-factors based on *F*^2^ are statistically about twice as large as those based on *F*, and *R*- factors based on ALL data will be even larger.

### Fractional atomic coordinates and isotropic or equivalent isotropic displacement parameters (Å^2^)

**Table:**

	*x*	*y*	*z*	*U*_iso_*/*U*_eq_	
Cl1	0.45135 (9)	1.22680 (17)	0.91318 (10)	0.0558 (4)	
N1	0.8522 (2)	0.8948 (4)	0.8966 (3)	0.0295 (8)	
N2	0.9299 (2)	0.8091 (4)	0.9689 (2)	0.0291 (8)	
H2	0.9246	0.7758	1.0291	0.035*	
O1	0.7409 (2)	1.0093 (4)	0.7040 (2)	0.0439 (8)	
H1	0.7853	0.9523	0.7442	0.066*	
O2	1.02312 (19)	0.8242 (4)	0.8572 (2)	0.0380 (8)	
O3	1.3085 (2)	0.4281 (4)	1.0233 (2)	0.0491 (9)	
H3	1.2983	0.4456	0.9592	0.074*	
O4	1.1915 (2)	0.5757 (4)	1.3090 (2)	0.0400 (8)	
H4	1.1481	0.6308	1.3229	0.060*	
O5	0.8783 (2)	0.7575 (4)	0.6646 (2)	0.0434 (8)	
H5A	0.9196	0.7761	0.7263	0.065*	
H5B	0.8435	0.6765	0.6750	0.065*	
C1	0.6935 (3)	1.0274 (5)	0.8653 (3)	0.0274 (9)	
C2	0.6769 (3)	1.0606 (5)	0.7568 (3)	0.0309 (10)	
C3	0.5923 (3)	1.1451 (6)	0.6983 (3)	0.0426 (12)	
H3A	0.5819	1.1666	0.6260	0.051*	
C4	0.5231 (3)	1.1979 (6)	0.7450 (4)	0.0437 (12)	
H4A	0.4662	1.2550	0.7050	0.052*	
C5	0.5392 (3)	1.1652 (6)	0.8520 (3)	0.0364 (11)	
C6	0.6229 (3)	1.0829 (5)	0.9126 (3)	0.0354 (11)	
H6	0.6330	1.0638	0.9851	0.042*	
C7	0.7800 (3)	0.9389 (5)	0.9314 (3)	0.0298 (10)	
H7	0.7838	0.9127	1.0016	0.036*	
C8	1.0139 (3)	0.7786 (5)	0.9438 (3)	0.0254 (9)	
C9	1.0938 (3)	0.6850 (5)	1.0242 (3)	0.0252 (9)	
C10	1.1629 (3)	0.6018 (5)	0.9869 (3)	0.0291 (10)	
H10	1.1574	0.6065	0.9145	0.035*	
C11	1.2398 (3)	0.5122 (5)	1.0569 (3)	0.0304 (10)	
C12	1.2492 (3)	0.5052 (5)	1.1645 (3)	0.0334 (10)	
H12	1.3011	0.4455	1.2117	0.040*	
C13	1.1801 (3)	0.5882 (5)	1.2015 (3)	0.0285 (10)	
C14	1.1025 (3)	0.6788 (5)	1.1327 (3)	0.0297 (10)	
H14	1.0570	0.7345	1.1587	0.036*	

### Atomic displacement parameters (Å^2^)

**Table:**

	*U*^11^	*U*^22^	*U*^33^	*U*^12^	*U*^13^	*U*^23^
Cl1	0.0391 (7)	0.0748 (10)	0.0587 (8)	0.0186 (7)	0.0226 (6)	−0.0009 (7)
N1	0.0256 (18)	0.032 (2)	0.0297 (19)	0.0018 (16)	0.0075 (15)	0.0043 (16)
N2	0.0262 (18)	0.039 (2)	0.0238 (17)	0.0072 (16)	0.0095 (15)	0.0089 (16)
O1	0.0415 (18)	0.061 (2)	0.0333 (17)	0.0121 (17)	0.0175 (15)	0.0069 (16)
O2	0.0337 (17)	0.057 (2)	0.0252 (15)	0.0057 (15)	0.0122 (13)	0.0097 (15)
O3	0.0467 (19)	0.069 (2)	0.0379 (18)	0.0283 (18)	0.0223 (16)	0.0028 (17)
O4	0.0422 (19)	0.053 (2)	0.0282 (16)	0.0143 (16)	0.0160 (14)	0.0059 (14)
O5	0.0438 (18)	0.058 (2)	0.0298 (16)	−0.0058 (17)	0.0131 (14)	−0.0021 (15)
C1	0.022 (2)	0.031 (3)	0.026 (2)	0.0015 (19)	0.0036 (17)	0.0020 (19)
C2	0.029 (2)	0.037 (3)	0.029 (2)	0.000 (2)	0.0129 (19)	0.000 (2)
C3	0.041 (3)	0.057 (3)	0.027 (2)	0.010 (2)	0.006 (2)	0.009 (2)
C4	0.034 (3)	0.050 (3)	0.042 (3)	0.012 (2)	0.004 (2)	0.009 (2)
C5	0.026 (2)	0.044 (3)	0.041 (3)	0.008 (2)	0.014 (2)	−0.002 (2)
C6	0.035 (2)	0.043 (3)	0.031 (2)	0.000 (2)	0.015 (2)	−0.001 (2)
C7	0.028 (2)	0.036 (3)	0.028 (2)	−0.003 (2)	0.0116 (18)	0.0002 (19)
C8	0.025 (2)	0.028 (2)	0.025 (2)	−0.0002 (19)	0.0087 (17)	0.0024 (18)
C9	0.024 (2)	0.029 (2)	0.025 (2)	−0.0001 (18)	0.0104 (17)	−0.0021 (18)
C10	0.031 (2)	0.034 (3)	0.025 (2)	0.002 (2)	0.0119 (18)	−0.0024 (19)
C11	0.029 (2)	0.035 (3)	0.028 (2)	0.005 (2)	0.0111 (19)	−0.002 (2)
C12	0.032 (2)	0.039 (3)	0.029 (2)	0.008 (2)	0.0097 (19)	0.003 (2)
C13	0.028 (2)	0.032 (3)	0.026 (2)	−0.001 (2)	0.0088 (18)	0.0033 (19)
C14	0.027 (2)	0.037 (3)	0.028 (2)	0.003 (2)	0.0145 (19)	−0.001 (2)

### Geometric parameters (Å, °)

**Table:**

Cl1—C5	1.746 (4)	C3—C4	1.372 (6)
N1—C7	1.290 (4)	C3—H3A	0.9300
N1—N2	1.386 (4)	C4—C5	1.376 (6)
N2—C8	1.348 (4)	C4—H4A	0.9300
N2—H2	0.8600	C5—C6	1.368 (6)
O1—C2	1.364 (4)	C6—H6	0.9300
O1—H1	0.8200	C7—H7	0.9300
O2—C8	1.238 (4)	C8—C9	1.484 (5)
O3—C11	1.362 (4)	C9—C10	1.391 (5)
O3—H3	0.8200	C9—C14	1.392 (5)
O4—C13	1.373 (4)	C10—C11	1.382 (5)
O4—H4	0.8200	C10—H10	0.9300
O5—H5A	0.8500	C11—C12	1.378 (5)
O5—H5B	0.8500	C12—C13	1.387 (5)
C1—C2	1.395 (5)	C12—H12	0.9300
C1—C6	1.401 (5)	C13—C14	1.386 (5)
C1—C7	1.442 (5)	C14—H14	0.9300
C2—C3	1.377 (6)		
			
C7—N1—N2	115.8 (3)	C1—C6—H6	120.0
C8—N2—N1	119.3 (3)	N1—C7—C1	122.3 (4)
C8—N2—H2	120.4	N1—C7—H7	118.8
N1—N2—H2	120.4	C1—C7—H7	118.8
C2—O1—H1	109.5	O2—C8—N2	121.6 (3)
C11—O3—H3	109.5	O2—C8—C9	121.8 (3)
C13—O4—H4	109.5	N2—C8—C9	116.6 (3)
H5A—O5—H5B	103.8	C10—C9—C14	119.7 (4)
C2—C1—C6	118.5 (4)	C10—C9—C8	116.8 (3)
C2—C1—C7	123.3 (4)	C14—C9—C8	123.4 (3)
C6—C1—C7	118.3 (4)	C11—C10—C9	120.4 (4)
O1—C2—C3	117.5 (4)	C11—C10—H10	119.8
O1—C2—C1	122.4 (4)	C9—C10—H10	119.8
C3—C2—C1	120.1 (4)	O3—C11—C12	117.6 (4)
C4—C3—C2	121.1 (4)	O3—C11—C10	122.1 (3)
C4—C3—H3A	119.5	C12—C11—C10	120.3 (4)
C2—C3—H3A	119.5	C11—C12—C13	119.2 (4)
C3—C4—C5	119.0 (4)	C11—C12—H12	120.4
C3—C4—H4A	120.5	C13—C12—H12	120.4
C5—C4—H4A	120.5	O4—C13—C14	121.5 (3)
C6—C5—C4	121.4 (4)	O4—C13—C12	117.2 (4)
C6—C5—Cl1	118.5 (3)	C14—C13—C12	121.4 (4)
C4—C5—Cl1	120.1 (3)	C13—C14—C9	119.0 (4)
C5—C6—C1	119.9 (4)	C13—C14—H14	120.5
C5—C6—H6	120.0	C9—C14—H14	120.5

### Hydrogen-bond geometry (Å, °)

**Table:**

*D*—H···*A*	*D*—H	H···*A*	*D*···*A*	*D*—H···*A*
O1—H1···N1	0.82	1.98	2.685 (4)	144
O1—H1···O5	0.82	2.47	2.952 (4)	119
O3—H3···O1^i^	0.82	2.10	2.916 (4)	173
O4—H4···O2^ii^	0.82	1.99	2.762 (4)	158
N2—H2···O5^ii^	0.86	2.09	2.931 (4)	164
O5—H5A···O2	0.85	1.91	2.760 (4)	174
O5—H5B···O4^iii^	0.85	2.11	2.902 (4)	156

Symmetry codes: (i) −*x*+2, *y*−1/2, −*z*+3/2; (ii) *x*, −*y*+3/2, *z*+1/2; (iii) −*x*+2, −*y*+1, −*z*+2.

## References

[bb1] Allen, F. H., Kennard, O., Watson, D. G., Brammer, L., Orpen, A. G. & Taylor, R. (1987). *J. Chem. Soc. Perkin Trans. 2*, pp. S1–19.

[bb2] Bhandari, S. V., Bothara, K. G., Raut, M. K., Patil, A. A., Sarkate, A. P. & Mokale, V. J. (2008). *Bioorg. Med. Chem.* **16**, 1822–1831. 10.1016/j.bmc.2007.11.014 18248993

[bb3] Herrick, R. S., Ziegler, C. J., Precopio, M., Crandall, K., Shaw, J. & Jarret, R. M. (2008). *J. Organomet. Chem.* **693**, 619–624.

[bb4] Jiang, Q.-H., Xu, Y.-H., Jian, L.-Y. & Zhao, L.-M. (2008). *Acta Cryst.* E**64**, o338. 10.1107/S1600536807067177 PMC2915379 21200899

[bb5] Sheldrick, G. M. (2008). *Acta Cryst.* A**64**, 112–122. 10.1107/S0108767307043930 18156677

[bb6] Siemens (1996). *SMART*, *SAINT* and *SADABS* Siemens Analytical X-ray Instruments Inc., Madison, Wisconsin, USA.

[bb7] Sinha, D., Tiwari, A. K., Singh, S., Shukla, G., Mishra, P., Chandra, H. & Mishra, A. K. (2008). *Eur. J. Med. Chem.* **43**, 160–165. 10.1016/j.ejmech.2007.03.022 17532543

[bb8] Suresh, P., Srimurugan, S. & Pati, H. N. (2007). *Chem. Lett.* **36**, 1332–1333.

